# Development and validation of a questionnaire to identify barriers to the implementation of the Clinical Practice Guidelines for lower limb amputees in a middle‐income country

**DOI:** 10.1002/gin2.12010

**Published:** 2024-03-19

**Authors:** Ana‐Maria Posada‐Borrero, Jesús Plata‐Contreras, Luz Helena Lugo‐Agudelo, Juan Carlos Velásquez‐Correa, Daniel F. Patiño‐Lugo, Maria del Pilar Pastor‐Durango, Daniel Camilo Aguirre‐Acevedo

**Affiliations:** ^1^ Health Rehabilitation Research Group, School of Medicine University of Antioquia Medellín Colombia

**Keywords:** barriers, diffusion of innovation, implementation science, practice guideline, psychometrics, surveys and questionnaires

## Abstract

**Aim:**

To develop and validate a questionnaire to identify the perceived barriers in the implementation of the Clinical Practice Guidelines for the lower limb amputee (CPG‐AMP).

**Study Design and Setting:**

The study consisted of two stages: first, the development of the questionnaire based on a meta‐review of the literature and interviews with patients and health providers (mixed‐methods research). Second, the evaluation of its psychometric properties was performed. Participants included health providers from hospitals and clinics, prosthetic workshops and academic institutions in Colombia.

**Results:**

A total of 90 items were obtained from the literature review and interviews. The validation of a preliminary 66‐item questionnaire was performed with 545 participants. After the factorial analysis, a 25‐item questionnaire with four domains was developed. Internal consistency was adequate in all domains, with Cronbach's *α* values between 0.76 and 0.83. Test–retest reliability in 58 participants yielded intraclass correlation coefficients between 0.51 and 0.59.

**Conclusions:**

A 25‐item questionnaire with four domains (health system, guideline, institutional and individual) was proposed to measure the perception of barriers to the CPG‐AMP. The conceptual framework and the questionnaire can be used to identify barriers of other CPGs and to help design strategies aimed at improving its implementation.

## Highlights

- Identifying implementation barriers of Clinical Practice Guidelines is encouraged.
- A 25‐item questionnaire with four domains was developed using mixed methods.
- A lack of dissemination of the Practice Guidelines was one of the major results.
- There is a need to design better strategies to improve the implementation process.

## 1 INTRODUCTION

Clinical Practice Guidelines (CPGs) are statements developed systematically to help professionals and patients make decisions about appropriate medical care for specific clinical circumstances.^1^ In Colombia, the CPGs for the diagnosis and preoperative, intraoperative and postoperative management, the prescription of the prosthesis and the integral rehabilitation of the amputated person (CPG‐AMP) were published by the Ministry of Health in 2016.^2^ However, there is no information about the implementation process or the factors affecting its implementation in the national territory.

The evidence‐based CPG Implementation Manual in Colombia recommends that the guidelines developing group should identify implementation barriers and facilitators during the development of CPG.^3^ The barriers to implementation are defined as those factors that can prevent, limit or hinder the implementation of the recommendations outlined in CPG; the facilitating factors are those that promote or favour changes.^3^ Because there is no direct way to measure these factors, perceived barriers can be thought of as latent variables or constructs, that is, an attribute that is not “operationally defined.”^4^

In the literature, different theoretical models have been proposed to identify barriers in the implementation of innovations in practice, without there being agreement on the number of domains and items of this construct.^5^, ^6^, ^7^, ^8^ The purpose of this study was to develop and validate a questionnaire to identify the barriers perceived by health providers in the implementation of the CPG‐AMP in Colombia.

## 2 METHODS

This study is part of a larger project that consisted of two phases: the development of the questionnaire and the evaluation of its psychometric properties. For the first phase, a systematic metareview and a mixed‐methods study were done. The detailed methodology and results of these two studies can be consulted elsewhere.^9^, ^10^ For the second phase, a cross‐sectional study to evaluate the questionnaire's psychometric properties was performed.

### 2.1 Development of the questionnaire

#### 2.1.1 Item selection

A search in PubMed, Embase, Cochrane Health Systems Evidence and International Guideline Network Library databases was performed. Systematic reviews that identified barriers or facilitators for the implementation of CPG were included.

Article selection and data extraction were carried out by two independent reviewers. The methodological quality of the studies was assessed using the Johanna Briggs Institute Checklist for Systematic Reviews and Research Syntheses.^11^ The synthesis was performed by qualitatively analysing the common and recurring elements and their frequencies.

Additionally, semistructured interviews were used to explore individual perceived barriers and facilitators to the implementation of guidelines for lower‐limb amputee patients and health providers in Colombia, using convenience sampling to ensure different perspectives.

With a first list of items, a preliminary questionnaire was elaborated and pilot‐tested to evaluate the validity of appearance, clarity and time needed to complete the responses.

#### 2.1.2 Pilot study

A pilot study was conducted with health providers to evaluate the response distribution, including the presence of a floor effect (more than 15% in the lowest response option) and a ceiling effect (more than 15% in the highest response option).^12^, ^13^ This information was then used to reduce the number of items in the questionnaire.

### 2.2 Evaluation of psychometric properties

#### 2.2.1 Sample size and participants

Information was collected between April 2018 and March 2019 from institutions providing health services of varying complexity, prosthetic workshops and academic institutions in Colombia. The participants were health providers, administrative staff and academic professionals who were contacted using a snowball sampling technique and by announcements to medical–scientific associations and social networks.

The sample size for structural construct validity^14^, ^15^, ^16^ and internal consistency^17^ included eight participants per item for a total of 545 participants. The following were assumed for the test–retest reliability: a type I error of 0.05 and a type II error of 0.2; intraclass correlation coefficient (ICC) for the null hypothesis of 0.6 and 0.8 for the alternative hypothesis; and 10% possible losses, for a total of 44 participants.^18^

#### 2.2.2 Procedure

Two questionnaire formats were used, one physical and other digital (Google forms®) including the same variables, item order and response options. The digital format was designed not to allow missing data. For the physical format, the personnel in charge of collection verified the quality of the records and the presence of missing data before delivering the questionnaires. When it was not possible to contact the participant to complete the data, imputation was performed using the average score of the item.^19^

#### 2.2.3 Content validity

A discussion session was held with five of the questionnaire developers as an item‐reduction exercise. The items were selected by consensus considering the clarity and importance of the item for the construct. Items with four or five votes in favour were included.

#### 2.2.4 Structural validity: Confirmatory factor analysis

Confirmatory factor analysis (CFA) was performed according to the domains proposed by Cabana et al.,^5^ Peters et al.,^6^ Flottorp et al.^7^ and Michie.^8^ For each theoretical model, the items of the preliminary questionnaire were distributed in the different domains. The goodness of fit was evaluated according to the following reference values: comparative fit index ≥ 0.95; Tucker–Lewis Index ≥ 0.95; root‐mean‐square error of approximation < 0.08; and weighted root‐mean‐square residual < 0.90.^20^

#### 2.2.5 Structural validity: Exploratory factor analysis

Exploratory factor analysis (EFA), was performed due to a poor fit to the theoretical models according to the CFA. Weighted least‐square mean and variance^21^, ^22^ with Geomin rotation^23^ was the estimation method used with MPLUS software.^24^ For factor selection, an eigenvalue greater than one was considered, and the factors had to contain at least three items with factor loadings greater than or equal to 0.4.^25^

#### 2.2.6 Internal consistency and test–retest reliability

Internal consistency was evaluated for each domain using Cronbach's *α*, and a coefficient greater than 0.70 was interpreted as reliable.^20^ The test–retest reliability was evaluated by readministering the questionnaire between 10 and 30 days after the initial evaluation.^19^, ^26^, ^27^ A composite score was calculated for each domain, based on the unweighted sum of the response to the items. The test‐retest reliability was evaluated using the ICC and the Bland and Altman plot.^28^, ^29^

#### 2.2.7 Statistical analysis

The items were described according to the frequency distribution of each response option, and measures of central tendency, dispersion, asymmetry and kurtosis were calculated. The analyses were performed in SPSS version 24.0.^30^

## 3 RESULTS

### 3.1 Development of the questionnaire

The research group obtained information from a literature metareview^9^ and from individual interviews with potential users of CPG‐AMP.^10^ Subsequently, preliminary tests were performed with health providers.

#### 3.1.1 Item selection

Ninety items were obtained from the information gathered from the literature metareview and individual interviews conducted with nine patients with lower limb amputation and 29 providers from health institutions.^9^, ^10^ With the list of 90 items the first version of the questionnaire was developed and pilot‐tested with 13 professionals. The mean application time was 28 min (range: 14–60). Of the 90 items, 17 were deleted, 33 had wording changes and 40 were left unchanged, resulting in a second questionnaire with 73 items (90‐ and 73‐item questionnaires in Spanish can be found in Appendices S1 and S2).

#### 3.1.2 Pilot study

**Figure 1:**
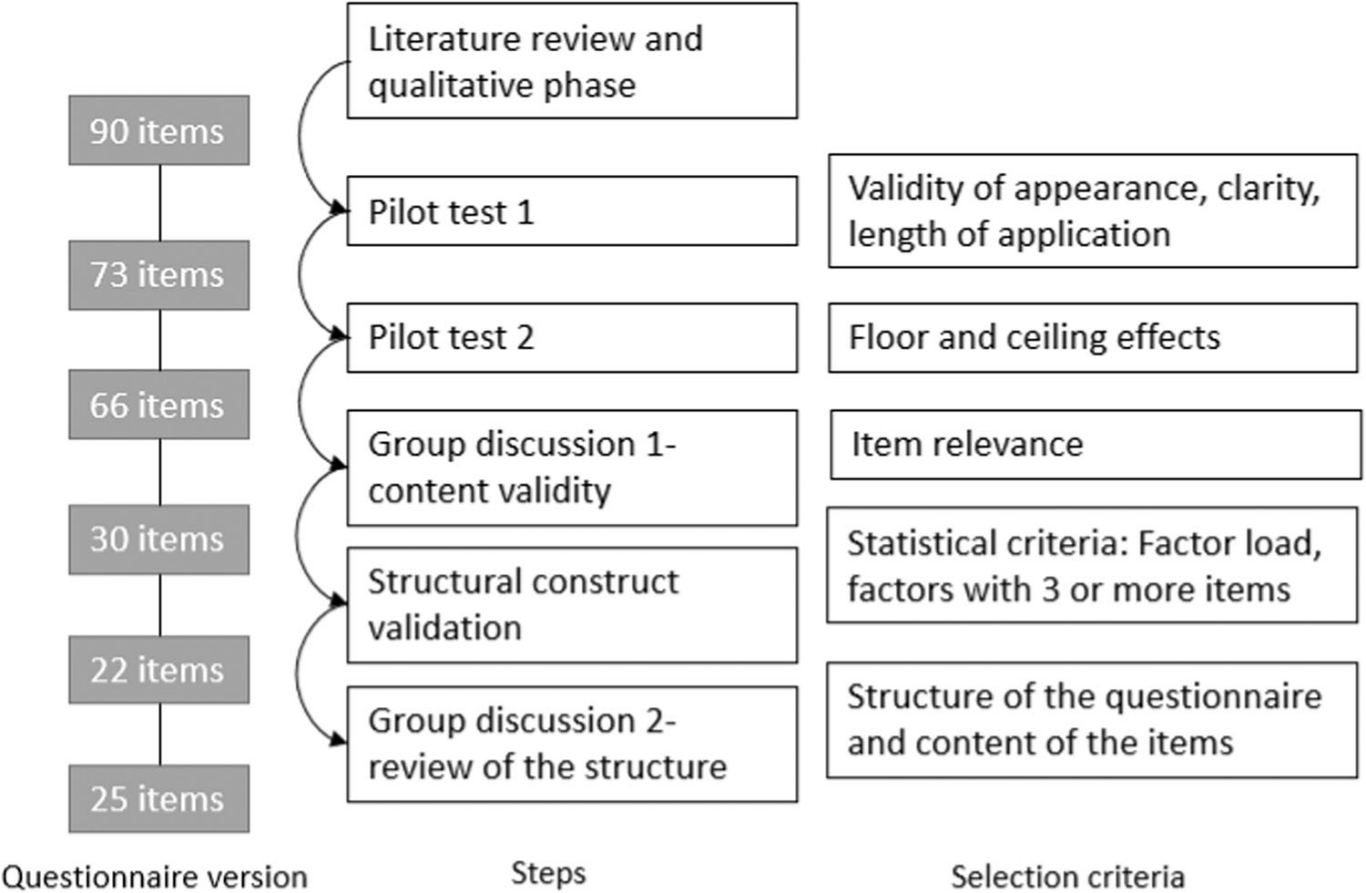
Development of the Clinical Practice Guidelines for the lower limb amputee barriers questionnaire.

A preliminary study was conducted with 136 participants with the second version of the questionnaire. In five of the 73 items, there was a ceiling effect, and in 66 items, there was a floor effect. Due to these findings, the questions were rephrased. Seven items were excluded because they were repeated or were contained in other questions, producing a new version with 66 items (see Figure 1). The time needed to complete the questionnaire was not measured in this second pilot study.

### 3.2 Psychometric properties

In this phase, 545 subjects participated, with a median age of 37 years (interquartile range [IQR]: 17) and with 10 years of experience in their field (IQR: 15.8). The general characteristics of this sample are presented in Table 1. Only 27% of the participants were aware of the CPG‐AMP. Three hundred and forty‐four completed the physical questionnaire, and 201 completed the digital questionnaire. In 12 questionnaires (2%), missing data were found, and simple imputation was performed using the mean score for each item.^19^

**Table 1:** Sociodemographic characteristics of the sample (*n* = 545).

Variable	Frequency (%)
Age (years), median (IQR)	37 (17)
Experience (years), median (IQR)	10 (15.8)
Education level	
Technologist/technician/student	17 (3.1)
Professional	528 (96.9)
Occupation	
Physical/occupational therapist	186 (34.1)
MD clinical specialty	158 (29.0)
MD surgical specialty	50 (9.2)
Director/manager/auditor	53 (9.7)
Nurse	35 (6.4)
Social worker/psychologist	23 (4.2)
Epidemiologist/researcher	16 (2.9)
Technologist/prosthetist	8 (1.5)
Other	16 (2.9)
Type of institution	
Health insurance company	25 (4.6)
Healthcare provider company	368 (67.6)
Academic institution	90 (16.5)
State public health company	13 (2.4)
Private office	25 (4.6)
Nongovernmental organization	8 (1.5)
Other	16 (2.9)
Level of complexity of the institution	
Low	31 (5.7)
Medium	206 (37.8)
High	243 (44.6)
Not applicable (academic institution)	65 (11.9)
Awareness of the CPG‐AMP	
Yes	146 (26.8)
No	399 (73.2)

Abbreviations: CPG‐AMP, Clinical Practice Guidelines for the lower limb amputee; IQR, interquartile range.

#### 3.2.1 Content validity

After the item‐reduction exercise, and according to the agreement index for the content validation process of the 66‐item version, 36 items were excluded, resulting in a new version with 30 items (see Figure 1).

#### 3.2.2 Structural validity: Confirmatory factor analysis

With the 66‐item version, structural validity was evaluated using CFA according to the domains proposed in previous theoretical models.^5^, ^6^, ^7^, ^8^ Table 2 presents the results that indicate a poor model fit.

**Table 2:** Goodness‐of‐fit indices of theoretical models and the new model according to the CFA.

Model	Description	*χ* ^2^	*df*	*χ* ^2^/*df*	*p* Value	CFI	TLI	RMSEA (90% CI)	WRMR
Cabana	Six domains	5339.0	804	6.64	<0.001	0.62	0.60	0.108 (0.105; 0.110)	3.11
Peters	Four domains	5349.3	813	6.58	<0.001	0.62	0.60	0.107 (0.104; 0.110)	3.16
Flottorp	Seven domains	5171.9	809	6.39	<0.001	0.64	0.61	0.105 (0.103; 0.108)	3.06
Michie	11 domains	5221.2	804	6.49	<0.001	0.63	0.61	0.106 (0.103; 0.109)	3.06

Abbreviations: CFA, confirmatory factor analysis; CFI, comparative fit index, ≥0.95; CI, confidence interval; df, degrees of freedom; RMSEA, root‐mean‐square error of approximation, <0.08; TLI, Tucker–Lewis index, ≥0.95; WRMR, weighted root‐mean‐square residual, <0.90^20^; *χ* ^2^/*df* ≤ 3.

#### 3.2.3 Structural validity: Exploratory factor analysis

EFA was performed with the 66‐item version, using the same data set from the CFA study. A principal component analysis on the 66‐item version for item reduction was done. Items with factor loadings less than 0.40 were excluded (items 1, 5, 15 and 19 of the 66‐item version), and factors with fewer than three items were rejected (items 8, 16, 54 and 55 of the 66‐item version) (see Appendix S3).

The final questionnaire included items derived from the EFA and an additional three items (Items 8, 14 and 16 or in the new numbering 2, 6 and 7) determined by the developer group given the importance to the construct. The domains worker context and worker behaviour were consolidated into a single domain called individual context.

Table 3 shows the final version of the questionnaire with 25 items, and Table 4 shows the four domains and the items that comprise them, with the means and medians of their scores.

**Table 3:** Questionnaire for the identification of barriers and facilitators of the implementation of the Clinical Practice Guidelines for the diagnosis and preoperative, intraoperative and postoperative treatment, the prescription of the prosthesis and the integral rehabilitation of the amputated person (CPG‐AMP‐B).

A. Please rate how much the following factors limit (or impede) the implementation of the CPG recommendations in your current clinical practice (mark only one answer)	Extremely	Very much	Neutral	Not much	Nothing
1. CPG training received by health professionals in universities	5	4	3	2	1
2. Nonacceptance of CPG recommendations by patients	5	4	3	2	1
3. Current awareness about the CPG by professionals at the institution	5	4	3	2	1
4. Applicability of CPG recommendations in practice	5	4	3	2	1
5. Change generated by the recommendations in routine clinical practice	5	4	3	2	1
6. Patient participation in the development of the CPG	5	4	3	2	1
7. Participation of patients and their families in decision‐making regarding their treatment	5	4	3	2	1
8. Existence of organized academic groups in the institution	5	4	3	2	1
9. Evidence‐based methodology of the CPG	5	4	3	2	1
10. Visual aids included in the CPG (charts, tables, flowcharts, etc.)	5	4	3	2	1
11. Disciplinary background of the CPG‐development group	5	4	3	2	1
12. Institutional monitoring of the implementation of the CPG	5	4	3	2	1

B. Please rate how often the following factors limit (or impede) the implementation of the CPG recommendations in your current clinical practice (mark only one answer)	Never	Rarely	Sometimes	Almost always	Always
13. Lack of human resources in the institution	1	2	3	4	5
14. Continuity in the process of patient care	1	2	3	4	5
15. Availability of technological resources in the institution	1	2	3	4	5
16. Recommended interventions not included in the benefit plan	1	2	3	4	5
17. Lack time for professionals to familiarize themselves with the CPG	1	2	3	4	5
18. Lack of a universal health information system in the country	1	2	3	4	5
19. Resistance to follow protocols by the professionals at the institution	1	2	3	4	5
20. Current timeliness of the authorization and administrative processes of health services	1	2	3	4	5
21. Lack of motivation by professionals to stay updated on the topics of the CPG	1	2	3	4	5
22. Lack of continuing education activities at the institution	1	2	3	4	5
23. Lack of access to health services in remote areas	1	2	3	4	5
24. Availability of supplies and medical devices at the institution	1	2	3	4	5
25. Teamwork of healthcare professionals at the institution	1	2	3	4	5

**Table 4:** Domains of the final questionnaire and the items comprising them.

Domain	Items	Median (IQR)	Mean (SD)
1 Individual context	p1 p2 p3 p4 p5 p7 p17 p19 p21	31 (8)	31.32 (5.78)
2 Institutional context	p8 p13 p15 p22 p24 p25	17 (6)	17.47 (4.75)
3 Health system context	p14 p16 p18 p20 p23	19 (5)	18.87 (3.80)
4 CPG context	p6 p9 p10 p11 p12	15 (8)	14.87 (5.05)
Total	25 items	83 (19)	82.53 (14.58)

Abbreviations: CPG, Clinical Practice Guidelines; IQR, interquartile range; SD, standard deviation.

The total score of the questionnaire was normally distributed. The mean score was 82.53 with a standard deviation of 14.58. No floor or ceiling effects were found for the domains or for the total score.

#### 3.2.4 Internal consistency and test–retest reliability

The test–retest reliability was evaluated by readministering the questionnaire to 58 participants. For the version with 25 items and four domains, Cronbach's *α* values were between 0.76 (domain: health system) and 0.83 (domain: CPG context) (Table 5). The ICC values for the domains of the 25‐item version, in the second application 10–30 days later, were between 0.51 (domain: institutional context) and 0.59 (domain: CPG context); the ICC for the total score was 0.60 (Table 5).

**Table 5:** Results of the internal consistency analysis (Cronbach's *α*) and test–retest reliability (ICC) for each CPG‐AMP barriers domain.

Domain	Items	Cronbach's *α* (CI_95_), *n* = 545	ICC (CI_95_), *n* = 58
1	Individual context	0.763 (0.732; 0.792)	0.585 (0.385; 0.732)
2	Institutional context	0.760 (0.728; 0.790)	0.509 (0.290; 0.677)
3	Health system context	0.798 (0.770; 0.824)	0.514 (0.299; 0.680)
4	CPG context	0.833 (0.810; 0.854)	0.589 (0.391; 0.735)
Total	25 items		0.602 (0.408; 0.744)

Abbreviations: CI, confidence interval; ICC, intraclass correlation coefficient.

**Figure 2:**
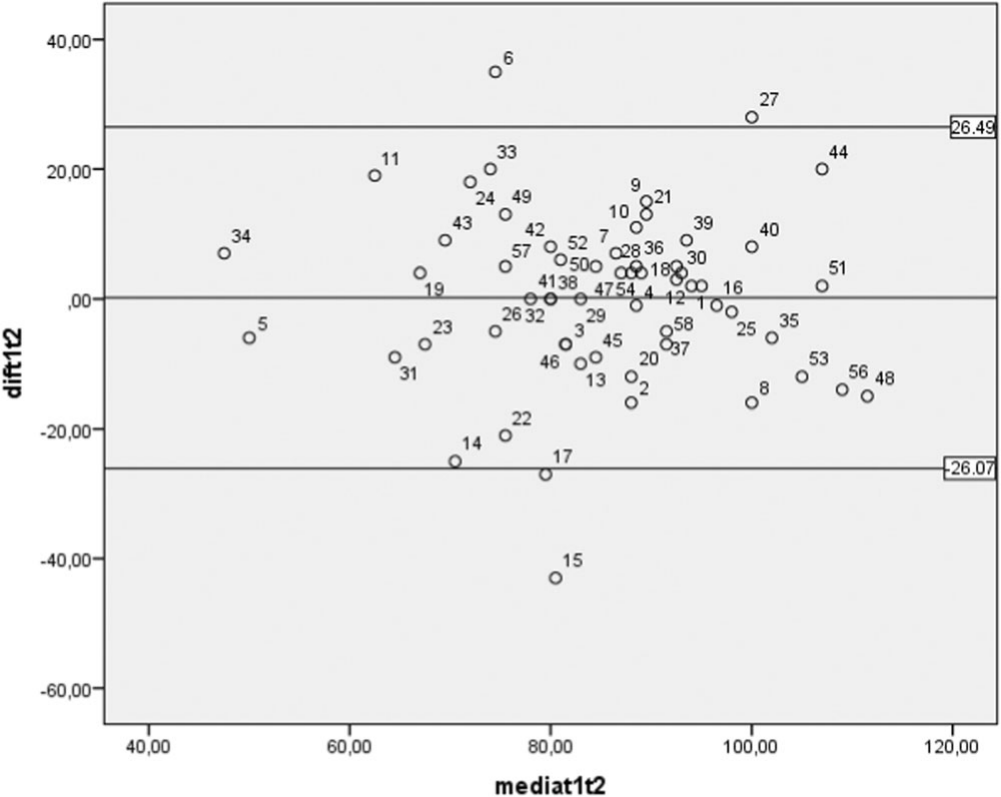
Bland and Altman plot showing the agreement between the two applications of the questionnaire for 58 participants.

The Bland and Altman plot (Figure 2) shows agreement between the first (t1) and the second application (t2) regarding the scores for the 58 participants.

## 4 DISCUSSION

The purpose of this study was to develop and validate a questionnaire to measure the perceived barriers to the implementation of the CPG‐AMP in healthcare providers. The items were obtained from a metareview of the literature and interviews with patients and health personnel.^9^, ^10^ To narrow down the items, qualitative and statistical procedures were used to select the most representative items of each domain. Using these techniques, the items were reduced from 90 to 25. Finally, the developers proposed a questionnaire with four domains and 25 items.

For content validity, items with four or more votes in favour of the developers were included. By using the indexes proposed in the literature,^31^, ^32^, ^33^ the number of items would have been significantly reduced. In the future, a greater number of evaluators should be considered for content validity analysis.

Structural validity was evaluated according to the domains proposed by Cabana et al.,^5^ Peters et al.,^6^ Flottorp et al.^7^ and Michie.^8^ The poor fit of the domains to the theoretical models likely occurred because these models are founded on expert consensus based on literature reviews that lack structural validity analysis. In addition, the majority of instruments are directed exclusively to medical personnel, without considering other health providers or patients.^34^

Regarding the domains of the proposed questionnaire, although some were included in previous theoretical models, the items that comprised them up are more specific to our context. For example, the individual domain was considered in all four models,^5^, ^6^, ^7^, ^8^ with items that refer to the awareness of the guideline and the lack of motivation of the professionals. However, they did not include items related to the lack of time for professionals to learn about the CPG and the training that professionals receive about evidence‐based medicine at universities.

The context of the health system was considered very briefly in the four models,^5^, ^6^, ^7^, ^8^ with items related to regulations and norms. However, aspects such as the absence of unified health information systems, the lack of access to health services in the most remote areas and the continuity in healthcare processes were not considered.

The domain related to the institutional context was also considered in the Peters,^6^ Flottorp^7^ and Michie^8^ models with items such as resource availability and teamwork. But only Flottorp et al.^7^ mentions items related to continuing education and academic groups. Finally, the domain of the context of the guideline is mentioned in the models of Cabana,^5^ Peters^6^ and Flottorp^7^ but the items they use do not correspond to those included in our questionnaire.

The reliability evaluation showed adequate internal consistency, with Cronbach's *α* values between 0.76 and 0.83 for the domains. The test–retest reliability in the 58 subjects who participated was between 0.51 and 0.59. Regarding the reliability studies of the theoretical models evaluated, only Peters et al.^6^ reported Cronbach's *α* values between 0.63 and 0.68, indicating low internal consistency, but did not mention test–retest analysis. Other instruments identifying specific implementation barriers to guidelines for hand hygiene,^35^ enteral nutrition^36^ and chronic obstructive pulmonary disease^37^ have reported internal consistency values between 0.66 and 0.92^35^, ^36^, ^37^ and test–retest values between 0.39 and 0.86,^35^, ^36^ a range in which the values of the present study are found.

### 4.1 Strengths

The proposed questionnaire was designed using mixed‐methods research, which included semistructured interviews and literature reviews for developing the items.

The validation sample included 545 participants, for a ratio of eight subjects per item, considering the number of items in the third version of the questionnaire (66 items), which was used for the validation process. The majority of the items had factor loadings above 0.60, and the factors had more than four items; therefore, the sample was adequate for the analyses.

Although there is no precise construct definition, the domains and items of the proposed questionnaire are similar to those of the models published in the literature,^5^, ^6^, ^7^, ^8^ which reinforces the theoretical concept of the perceived barriers and defines the construct as multidimensional.

Another strength of the study is that several areas of medicine were included: clinical and surgical; academic and administrative; technical and professional. Likewise, the different levels of care complexity in the main cities of the country were taken into account.

It is important to clarify that the questionnaire proposed in this study is based on an analysis of structural validity and contains items that can be used to assess perceived barriers to other CPG, not just the guidelines for individuals with lower limb amputations. The theoretical model proposed in this study may be used in other areas and published guidelines to measure perceived barriers to implementation.

### 4.2 Limitations

In the process to develop the questionnaire, several versions emerged, but the validation of the psychometric properties was done only with the 66‐ and 30‐item versions. The time to administer the questionnaire was only measured with the first version of the instrument. Due to the small sample size for evaluating the test–retest reliability at 10 days, the time frame for the second application was increased to 30 days. This could have affected the perception of barriers due to acquiring new knowledge, such as having reviewed the CPG‐AMP, which could have led to low ICC values for several of the participants.

### 4.3 Implications for practice

A lack of knowledge of the CPG‐AMP was one of the most important findings of this study, as it was found that the majority of participants were not aware of the guidelines. This probably reflects the lack of dissemination of these CPG and reinforces the need to design better strategies to improve this process. While there are individual efforts in our country to implement CPG, a structured national policy is needed to achieve this objective, and studies such as this could be used as a diagnostic tool to guide and design suitable implementation strategies.

## 5 CONCLUSIONS

Mixed methods were used to develop and validate a questionnaire to identify the perceived barriers in the implementation of the CPGs for the lower limb amputee in Colombia. The 25 items included in the questionnaire are distributed in four domains, related to health system; guidelines; institutional and individual contexts. The conceptual framework can be used for other studies that aim to understand barriers to the implementation of CPG in other health areas. Additional studies are needed to evaluate the performance of this questionnaire as a tool in the evaluation and implementation strategies of other CPGs in healthcare institutions.

## AUTHOR CONTRIBUTIONS

*Study conception and design*: Ana‐Maria Posada‐Borrero, Luz Helena Lugo‐Agudelo, Jesús Plata‐Contreras, Daniel F. Patiño‐Lugo, Maria del Pilar Pastor‐Durango and Daniel Camilo Aguirre‐Acevedo. *Data collection*: Ana‐Maria Posada‐Borrero and Juan Carlos Velásquez‐Correa. *Analysis and interpretation of results*: Ana‐Maria Posada‐Borrero, Daniel Camilo Aguirre‐Acevedo and Juan Carlos Velásquez‐Correa. *Draft manuscript preparation*: Ana‐Maria Posada‐Borrero. All authors reviewed the results and approved the final version of the manuscript.

## CONFLICT OF INTEREST STATEMENT

The authors declare no conflict of interest.

## ETHICS STATEMENT

All methods in the study were performed in accordance with the Declarations of Helsinki on ethical principles for medical research involving human subjects and it was considered as minimal risk. The study was approved by the Bioethics Committee of the Medical Research Institute of the University of Antioquia (Certificate of approval number 021, of 1 November 2017). For participants who completed the physical questionnaire, written informed consents were obtained. For the participants who filled out the digital questionnaires, the Bioethics Committee approved the use of the data if the participants checked the box in which they agreed to participate in the research. Also, we obtained the approval of the ethics committees in the participating health institutions.

## Supporting information

Supporting information.

Supporting information.

Supporting information.

## Data Availability

The data sets used and analysed during the current study are available from the corresponding author upon reasonable request.
