# Supplementary material for: Development and validation of a questionnaire to identify barriers to the implementation of the Clinical Practice Guidelines for lower limb amputees in a middle‐income country
Source: Clin Public Health Guidel. 2024 Mar 19;1(1):e12010. doi: 10.1002/gin2.12010 (PMC13563403; doi:10.1002/gin2.12010)
Supplement: Supplementary file 1 [file GIN2-1-e12010-s001.pdf]

FACULTAD DE MEDICINA - UNIVERSIDAD DE ANTIOQUIA

GRUPO DE REHABILITACION EN SALUD

Instrumento para la identificación de barreras y facilitadores de la implementación de la Guía de Práctica Clínica (GPC) para el diagnóstico y tratamiento preoperatorio, intraoperatorio y postoperatorio de la persona amputada, la prescripción de la prótesis y la rehabilitación integral

El propósito de este instrumento es identificar los factores que facilitan o dificultan el uso de la GPC. La información personal de los participantes será confidencial y los resultados del estudio sólo se utilizarán con fines investigativos.

**Datos del participante:**	1. Consecutivo:	2. Hora de inicio:	3. Hora final:
4. Fecha:	5. Cédula:	6. Edad:	
7. Ocupación: ☐ 1 Fisiatra<br>☐ 2 Ortopedista<br>☐ 3 Cirujano<br>☐ 4 Terapeuta físico<br>☐ 5 Terapeuta ocupacional<br>☐ 6 Protesista<br>☐ 7 Director/Gerente<br>☐ 8 Auditor<br>☐ 9 Trabajador social<br>☐10 Otro__________________	8. Tipo de institución: ☐ 1 EPS<br>☐ 2 IPS<br>☐ 3 Académica<br>☐ 4 ESE/Secretaría Salud<br>☐ 5 Consultorio particular<br>☐ 6 ONG<br>☐ 7 Otro______________	9. Ciudad: ☐ 1 Bogotá<br>☐ 2 Cali<br>☐ 3 Medellín<br>☐ 4 Santa Marta<br>☐ 5 Bucaramanga<br>☐ 6 Manizales<br>☐ 7 Otra _____________	
	10. Nivel de complejidad: ☐ 1 Bajo<br>(Si aplica) ☐ 2 Medio<br>☐ 3 Alto	11. Tiempo de experiencia:<br>_______ Años _______Meses	
12. ¿Conoce usted esta guía? 1 ☐Si 2 ☐No **(Nota: Aunque no la conozca puede continuar respondiendo la encuesta desde su percepción o experiencia).**			

A continuación se presenta una serie de 73 condiciones que pueden afectar la implementación de las recomendaciones de la GPC en estudio. Usted deberá evaluar en **qué grado** cada condición **facilita** la implementación de la GPC, en una escala de seis puntos, desde totalmente en desacuerdo hasta totalmente de acuerdo. Tenga en cuenta que puede seleccionar una de las seis opciones de acuerdo con la importancia que el enunciado tenga como barrera o como facilitador. Al final podrá proponer otros facilitadores que no se hayan incluido en este instrumento.

Ejemplo: Por favor califique, según su experiencia o su percepción, en **qué grado** está de acuerdo o no con que la difusión de la GPC **facilita** la implementación.

Totalmente en desacuerdo	Moderadamente en desacuerdo	Un poco en desacuerdo	Un poco de acuerdo	Moderadamente de acuerdo	Totalmente de acuerdo
1	2	3	✖4	5	6

Barrera ⟷ **Facilitador**

gruporehabilitacionsalud@udea.edu.co. Teléfono: 219 64 75. Dirección: Calle 62 52-59, Sede de Investigación Universitaria -SIU Torre 1, oficina 313. Medellín, Colombia.

Por favor califique, según su experiencia o su percepción, en **qué grado** está de acuerdo o no en que las siguientes condiciones **facilitan** la implementación de la GPC. Elija una sola opción por cada pregunta.

	Grado de acuerdo					
**Condición**	Totalmente en desacuerdo	Moderadamente en desacuerdo	Un poco en desacuerdo	Un poco de acuerdo	Moderadamente de acuerdo	Totalmente de acuerdo
1. La integración en la atención entre los niveles de baja, media y alta complejidad.	1	2	3	4	5	6
2. El alto impacto en el presupuesto de la institución para adoptar las recomendaciones de la GPC.	1	2	3	4	5	6
3. Tener disponibilidad de recursos humanos en su institución.	1	2	3	4	5	6
4. Tener disponibilidad de recursos tecnológicos para diagnóstico, intervención y rehabilitación en su institución.	1	2	3	4	5	6
5. La participación de la familia del paciente en la toma de decisiones sobre su tratamiento.	1	2	3	4	5	6
6. La existencia de asociaciones de pacientes o usuarios.	1	2	3	4	5	6
7. La formación académica en GPC de los profesionales en salud.	1	2	3	4	5	6
8. La afiliación a otros sistemas de aseguramiento en salud (SOAT, ARL).	1	2	3	4	5	6
9. El modelo del sistema general de seguridad social en salud.	1	2	3	4	5	6
10. La participación de las empresas comerciales en la elaboración de la GPC.	1	2	3	4	5	6
11. El conocimiento de los pacientes sobre la GPC.	1	2	3	4	5	6
12. La comunicación efectiva entre profesionales y pacientes.	1	2	3	4	5	6
13. La aceptación de las recomendaciones de la GPC por parte de los pacientes.	1	2	3	4	5	6
14. El conocimiento de los profesionales sobre la GPC.	1	2	3	4	5	6
15. La claridad de las recomendaciones de la GPC.	1	2	3	4	5	6

(Verifique que haya diligenciado todos los ítems. El instrumento está impreso por ambas caras del papel.)

Por favor califique, según su experiencia o su percepción, en **qué grado** está de acuerdo o no en que las siguientes condiciones **facilitan** la implementación de la GPC. Elija una sola opción por cada pregunta.

	Grado de acuerdo					
**Condición**	**Totalmente en desacuerdo**	**Moderadamente en desacuerdo**	**Un poco en desacuerdo**	**Un poco de acuerdo**	**Moderadamente de acuerdo**	**Totalmente de acuerdo**
16. La aplicabilidad de las recomendaciones de la GPC en la práctica.	1	2	3	4	5	6
17. La importancia de los desenlaces (Ej. funcionamiento, calidad de vida, capacidad de marcha) de las recomendaciones de la GPC.	1	2	3	4	5	6
18. La continuidad de la atención de los pacientes en la misma IPS.	1	2	3	4	5	6
19. La presencia de comorbilidades en los pacientes.	1	2	3	4	5	6
20. La inclusión de las intervenciones recomendadas en el plan de beneficios en salud.	1	2	3	4	5	6
21. La comunicación efectiva entre profesionales.	1	2	3	4	5	6
22. La costo-efectividad de las recomendaciones.	1	2	3	4	5	6
23. El cambio que generan las recomendaciones en la práctica clínica habitual.	1	2	3	4	5	6
24. La pertinencia del tema de la GPC para la institución.	1	2	3	4	5	6
25. El tiempo disponible de los profesionales para implementar las recomendaciones de la GPC.	1	2	3	4	5	6
26. El conocimiento del personal administrativo sobre la GPC.	1	2	3	4	5	6
27. La continuidad en la atención del paciente con el mismo profesional de la salud.	1	2	3	4	5	6
28. La participación de los pacientes en la elaboración de la GPC.	1	2	3	4	5	6
29. Los cambios frecuentes en la normatividad en el sistema de salud.	1	2	3	4	5	6
30. La participación activa de los pacientes en la toma de decisiones sobre su tratamiento.	1	2	3	4	5	6

(Verifique que haya diligenciado todos los ítems. El instrumento está impreso por ambas caras del papel.)

Por favor califique, según su experiencia o su percepción, en **qué grado** está de acuerdo o no en que las siguientes condiciones **facilitan** la implementación de la GPC. Elija una sola opción por cada pregunta.

	Grado de acuerdo					
**Condición**	**Totalmente en desacuerdo**	**Moderadamente en desacuerdo**	**Un poco en desacuerdo**	**Un poco de acuerdo**	**Moderadamente de acuerdo**	**Totalmente de acuerdo**
31. La afiliación al régimen contributivo en salud.	1	2	3	4	5	6
32. La aplicación de las recomendaciones en pacientes mayores a 60 años.	1	2	3	4	5	6
33. La aplicabilidad de las recomendaciones en pacientes de diferentes culturas o minorías étnicas.	1	2	3	4	5	6
34. La existencia de grupos académicos organizados en su institución.	1	2	3	4	5	6
35. El apoyo a los pacientes por parte de ONG, corporaciones o empresas sociales.	1	2	3	4	5	6
36. La afiliación a programas de Medicina prepagada o pólizas de aseguramiento.	1	2	3	4	5	6
37. El desarrollo o adopción simultáneo de otras GPC del mismo tema en su institución.	1	2	3	4	5	6
38. La afiliación al régimen subsidiado en salud.	1	2	3	4	5	6
39. La GPC como requisito para habilitación de la IPS.	1	2	3	4	5	6
40. El trabajo en equipo entre los proveedores de prótesis y los profesionales responsables de la atención de los pacientes.	1	2	3	4	5	6
41. El reconocimiento académico del grupo desarrollador de la GPC.	1	2	3	4	5	6
42. Los objetivos y el alcance de la GPC.	1	2	3	4	5	6
43. La afiliación a regímenes especiales (Fuerzas Armadas, Ecopetrol, Magisterio, Poder legislativo).	1	2	3	4	5	6
44. La resistencia por parte de los profesionales a seguir protocolos.	1	2	3	4	5	6
45. La agilidad del proceso de autorización de los servicios de salud.	1	2	3	4	5	6

(Verifique que haya diligenciado todos los ítems. El instrumento está impreso por ambas caras del papel.)

Por favor califique, según su experiencia o su percepción, en **qué grado** está de acuerdo o no en que las siguientes condiciones **facilitan** la implementación de la GPC. Elija una sola opción por cada pregunta.

	Grado de acuerdo					
**Condición**	**Totalmente en desacuerdo**	**Moderadamente en desacuerdo**	**Un poco en desacuerdo**	**Un poco de acuerdo**	**Moderadamente de acuerdo**	**Totalmente de acuerdo**
46. Las competencias clínicas, quirúrgicas y de rehabilitación de los profesionales.	1	2	3	4	5	6
47. La estandarización del proceso de atención por medio de la GPC.	1	2	3	4	5	6
48. Las buenas condiciones socioeconómicas de los pacientes.	1	2	3	4	5	6
49. El conocimiento del paciente acerca de su enfermedad.	1	2	3	4	5	6
50. La motivación del profesional para mantenerse actualizado en el tema de la GPC.	1	2	3	4	5	6
51. La cercanía de la residencia del paciente a los sitios de atención.	1	2	3	4	5	6
52. Los trámites administrativos dispendiosos para la remisión de pacientes entre instituciones.	1	2	3	4	5	6
53. Tener disponibilidad de insumos y dispositivos médicos en su institución.	1	2	3	4	5	6
54. Tener disponibilidad de los servicios de atención necesarios en su institución.	1	2	3	4	5	6
55. El trabajo en equipo de los profesionales asistenciales en su institución.	1	2	3	4	5	6
56. La información apropiada al paciente acerca del proceso de atención.	1	2	3	4	5	6
57. La participación de los profesionales en actividades de educación continua.57.	1	2	3	4	5	6
58. La metodología de la GPC basada en evidencia.	1	2	3	4	5	6
59. Tener disponibilidad de un sistema de información e indicadores en su institución.	1	2	3	4	5	6
60. El conocimiento de los profesionales sobre la medicina basada en la evidencia.	1	2	3	4	5	6

(Verifique que haya diligenciado todos los ítems. El instrumento está impreso por ambas caras del papel.)

Por favor califique, según su experiencia o su percepción, en **qué grado** está de acuerdo o no en que las siguientes condiciones **facilitan** la implementación de la GPC. Elija una sola opción por cada pregunta.

	Grado de acuerdo					
**Condición**	**Totalmente en desacuerdo**	**Moderadamente en desacuerdo**	**Un poco en desacuerdo**	**Un poco de acuerdo**	**Moderadamente de acuerdo**	**Totalmente de acuerdo**
61. La extensión o longitud del documento de la GPC.	1	2	3	4	5	6
62. Las ayudas visuales de las recomendaciones de la GPC (Gráficas, tablas, flujogramas, etc).	1	2	3	4	5	6
63. La terminología de la GPC (Calidad de la evidencia y fuerza de las recomendaciones).	1	2	3	4	5	6
64. La multidisciplinariedad de los desarrolladores de la guía.	1	2	3	4	5	6
65. El seguimiento institucional a la aplicación de la GPC.	1	2	3	4	5	6
66. La difusión de la GPC.	1	2	3	4	5	6
67. La inclusión de la GPC en la inducción institucional de los profesionales.	1	2	3	4	5	6
68. Trabajar en un hospital universitario.	1	2	3	4	5	6
69. La credibilidad de las GPC nacionales.	1	2	3	4	5	6
70. La formación en GPC que reciben los profesionales por parte de EPS e IPS.	1	2	3	4	5	6
71. La disminución de la variabilidad clínica no justificada al aplicar las recomendaciones de la GPC.	1	2	3	4	5	6
72. La aceptación de las recomendaciones de la GPC por parte del personal de la salud.	1	2	3	4	5	6
73. La pertinencia de la GPC en municipios pequeños	1	2	3	4	5	6

*Municipios pequeños: <15000 habitantes y bajos ingresos. Ejemplos: En **Antioquia**: Liborina; en **Atlántico**: Sabanalarga; en **Cauca**: Corinto; en **Cundinamarca**: Tocaima; en **Magdalena**: El banco; en **Norte de Santander**: Tibú; en **Quindío**: Salento; en **Risaralda**: La Virginia; en **Santander**: Málaga; en **Valle del Cauca**: Dagua

¿Qué otros factores considera usted como facilitadores para implementar la guía?:

____

____

¡Gracias por su participación!
