# Supplementary material for: Development and validation of a questionnaire to identify barriers to the implementation of the Clinical Practice Guidelines for lower limb amputees in a middle‐income country
Source: Clin Public Health Guidel. 2024 Mar 19;1(1):e12010. doi: 10.1002/gin2.12010 (PMC13563403; doi:10.1002/gin2.12010)
Supplement: Supplementary file 2 [file GIN2-1-e12010-s002.pdf]

Instrumento para la identificación de barreras y facilitadores de la implementación de guías de práctica clínica (GPC)

El propósito de este instrumento es identificar los factores que facilitan o dificultan el uso de la GPC. La información personal de los participantes será confidencial y los resultados del estudio sólo se utilizarán con fines investigativos.

Cédula: ___________________ Ciudad: 1.☐ Bogotá 2. ☐ Cali 3.☐ Medellín 4.☐ Santa Marta 5. ☐ Otra________________ Edad: _______ (Años)

Participante: 1.☐ Tecnólogo 2.☐ Profesional asistencial 3.☐ Administrativo 5. ☐ Otro____________________ Tiempo de experiencia: ____________ (Años)

Profesional: 1.☐ Fisiatra 2. ☐ Ortopedista 3.☐ Cirujano 4.☐ Terapeuta físico 5. ☐ Terapeuta ocupacional 6.☐ Otro ________________________________

Nivel de complejidad de la institución (Si aplica): 1.☐ Baja 2. ☐ Media 3. ☐ Alta Institución: ____

Tipo de institución: 1.☐ EPS 2. ☐ IPS 3. ☐ Académica 4.☐ Secretaría de salud 5. ☐ Consultorio particular 6.☐ ONG 5. ☐ Otro___________________

GPC a evaluar: **Guía de Práctica Clínica para el diagnóstico y tratamiento preoperatorio, intraoperatorio y postoperatorio de la persona amputada, la prescripción de la prótesis y la rehabilitación integral**
(http://gpc.minsalud.gov.co/gpc_sites/Repositorio/Conv_637/GPC_amputacion/gpc_amputacion_completa.aspx)

¿Usted conoce esta GPC? 1. ☐ Si 2. ☐ No (Nota: Aunque no la conozca puede continuar respondiendo la encuesta)

A continuación se presenta una serie de condiciones que pueden afectar la implementación de las recomendaciones de la GPC en estudio. Usted deberá evaluar si cada condición facilita la implementación de la GPC, en una escala de seis puntos, desde totalmente en desacuerdo hasta totalmente de acuerdo. Posteriormente podrá proponer otras barreras o facilitadores que no se hayan incluido en este instrumento.

Por favor califique, según su experiencia o su percepción, en qué grado las siguientes condiciones **facilitan** la implementación de la GPC.

Ítem	Totalmente en desacuerdo		Moderadamente en desacuerdo		Un poco en desacuerdo		Un poco de acuerdo		Moderadamente de acuerdo		Totalmente de acuerdo	
1. La articulación entre diferentes niveles de atención.	1		2		3		4		5		6	
2. El costo para la institución de adoptar las recomendaciones de la GPC.	1		2		3		4		5		6	
3. La actualización frecuente de la tecnología.	1		2		3		4		5		6	
4. La disponibilidad de recursos humanos en su institución.	1		2		3		4		5		6	
5. La disponibilidad de recursos tecnológicos para diagnóstico, intervención y rehabilitación en su institución.	1		2		3		4		5		6	
6. La participación de la familia del paciente en la toma de decisiones sobre su tratamiento.	1		2		3		4		5		6	
7. La existencia de asociaciones de pacientes o usuarios.	1		2		3		4		5		6	
8. La formación académica en GPC de los profesionales en salud.	1		2		3		4		5		6	
9. La afiliación a otros sistemas de aseguramiento en salud (SOAT, ARL).	1		2		3		4		5		6	
10. La organización del sistema de salud.	1		2		3		4		5		6	
11. La participación de las empresas comerciales en la elaboración de la GPC.	1		2		3		4		5		6	
12. El conocimiento de los pacientes sobre la GPC.	1		2		3		4		5		6	
13. La buena comunicación entre profesionales y pacientes.	1		2		3		4		5		6	
14. La aceptación de las recomendaciones de la GPC por parte de los pacientes.	1		2		3		4		5		6	
15. El conocimiento de los profesionales sobre la GPC.	1		2		3		4		5		6	

Por favor califique, según su experiencia o su percepción, en qué grado las siguientes condiciones **facilitan** la implementación de la GPC.

Ítem	Totalmente en desacuerdo		Moderadamente en desacuerdo		Un poco en desacuerdo		Un poco de acuerdo		Moderadamente de acuerdo		Totalmente de acuerdo	
16. La claridad de las recomendaciones de la GPC.	1		2		3		4		5		6	
17. La aplicabilidad de las recomendaciones de la GPC en la práctica.	1		2		3		4		5		6	
18. La importancia de los desenlaces (Ej. funcionamiento, calidad de vida, capacidad de marcha) de las recomendaciones de la GPC.	1		2		3		4		5		6	
19. La continuidad de la atención de los pacientes en las IPS.	1		2		3		4		5		6	
20. La actitud del paciente sobre su condición de salud.	1		2		3		4		5		6	
21. La aplicación de las recomendaciones a pacientes con diferentes condiciones de salud.	1		2		3		4		5		6	
22. La inclusión de las intervenciones recomendadas en el plan de beneficios.	1		2		3		4		5		6	
23. La buena comunicación entre profesionales.	1		2		3		4		5		6	
24. Los beneficios de implementar las recomendaciones superan los costos.	1		2		3		4		5		6	
25. Las recomendaciones que cambian la práctica clínica habitual.	1		2		3		4		5		6	
26. La pertinencia del tema de la GPC para la institución.	1		2		3		4		5		6	
27. El tiempo disponible para implementar cambios en el trabajo.	1		2		3		4		5		6	
28. El conocimiento del personal administrativo sobre la GPC.	1		2		3		4		5		6	
29. La continuidad en la atención con el mismo profesional de la salud.	1		2		3		4		5		6	
30. La participación de los pacientes en la construcción de la GPC.	1		2		3		4		5		6	

gruporehabilitacionsalud@udea.edu.co. Teléfono: 219 64 75. Dirección: Calle 62 52-59, Sede de Investigación Universitaria -SIU Torre 1, oficina 313. Medellín, Colombia.

Por favor califique, según su experiencia o su percepción, en qué grado las siguientes condiciones **facilitan** la implementación de la GPC.

	Totalmente en desacuerdo		Moderadamente en desacuerdo		Un poco en desacuerdo		Un poco de acuerdo		Moderadamente de acuerdo		Totalmente de acuerdo	
31. Los cambios frecuentes en la normatividad en el sistema de salud.	1		2		3		4		5		6	
32. La importancia de las GPC para la institución.	1		2		3		4		5		6	
33. La participación de pacientes en la toma de decisiones sobre su tratamiento.	1		2		3		4		5		6	
34. La afiliación al régimen contributivo en salud.	1		2		3		4		5		6	
35. La aplicación de las recomendaciones en pacientes adultos mayores.	1		2		3		4		5		6	
36. El impacto de las recomendaciones en la práctica clínica.	1		2		3		4		5		6	
37. La aplicabilidad de las recomendaciones en pacientes de diferentes culturas.	1		2		3		4		5		6	
38. La existencia de grupos académicos organizados en su institución.	1		2		3		4		5		6	
39. El apoyo a los pacientes por parte de ONG, corporaciones o empresas sociales.	1		2		3		4		5		6	
40. La afiliación a programas de Medicina prepagada o pólizas de aseguramiento.	1		2		3		4		5		6	
41. El desarrollo o adopción de otras GPC del mismo tema en su institución.	1		2		3		4		5		6	
42. La afiliación al régimen subsidiado en salud.	1		2		3		4		5		6	
43. La GPC como requisito para habilitación de la IPS.	1		2		3		4		5		6	
44. Las comorbilidades del paciente.	1		2		3		4		5		6	
45. La articulación entre los proveedores de prótesis y los profesionales responsables de la atención de los pacientes.	1		2		3		4		5		6	

Por favor califique, según su experiencia o su percepción, en qué grado las siguientes condiciones **facilitan** la implementación de la GPC.

	Totalmente en desacuerdo		Moderadamente en desacuerdo		Un poco en desacuerdo		Un poco de acuerdo		Moderadamente de acuerdo		Totalmente de acuerdo	
46. El reconocimiento del grupo desarrollador de la GPC.	1		2		3		4		5		6	
47. La experiencia del profesional en el campo de interés de la GPC.	1		2		3		4		5		6	
48. El tiempo para estar informado sobre nuevas GPC.	1		2		3		4		5		6	
49. Los objetivos y el alcance de la GPC.	1		2		3		4		5		6	
50. La afiliación a regímenes especiales (Fuerzas Armadas, Ecopetrol, Magisterio, Poder legislativo).	1		2		3		4		5		6	
51. La resistencia de los profesionales a seguir protocolos.	1		2		3		4		5		6	
52. El proceso de autorización de los servicios de salud.	1		2		3		4		5		6	
53. Las competencias clínicas, quirúrgicas y de rehabilitación de los profesionales.	1		2		3		4		5		6	
54. La estandarización de la atención por medio de la GPC.	1		2		3		4		5		6	
55. Las buenas condiciones socioeconómicas de los pacientes.	1		2		3		4		5		6	
56. El conocimiento del paciente acerca de su enfermedad.	1		2		3		4		5		6	
57. La motivación del profesional para mantenerse actualizado en el tema de la GPC.	1		2		3		4		5		6	
58. El apoyo a los profesionales por parte del área administrativa de la institución.	1		2		3		4		5		6	
59. La residencia del paciente cerca de los sitios de atención.	1		2		3		4		5		6	
60. El funcionamiento de las redes integradas de servicios.	1		2		3		4		5		6	

Por favor califique, según su experiencia o su percepción, en qué grado las siguientes condiciones **facilitan** la implementación de la GPC.

	Totalmente en desacuerdo		Moderadamente en desacuerdo		Un poco en desacuerdo		Un poco de acuerdo		Moderadamente de acuerdo		Totalmente de acuerdo	
61. La forma como están redactadas las recomendaciones de la GPC.	1		2		3		4		5		6	
62. Los trámites administrativos para el traslado de pacientes entre instituciones.	1		2		3		4		5		6	
63. La disponibilidad de insumos y dispositivos médicos en su institución.	1		2		3		4		5		6	
64. La disponibilidad de los servicios de atención necesarios en su institución.	1		2		3		4		5		6	
65. El trabajo en equipo en su institución.	1		2		3		4		5		6	
66. El conocimiento del paciente acerca del proceso de atención.	1		2		3		4		5		6	
67. La pertinencia de la GPC en el contexto municipal.	1		2		3		4		5		6	
68. La GPC basada en evidencia.	1		2		3		4		5		6	
69. La disponibilidad de un sistema de información e indicadores en su institución.	1		2		3		4		5		6	
70. El reconocimiento de la utilidad de la GPC por parte de los pacientes.	1		2		3		4		5		6	
71. La disponibilidad de un equipo interdisciplinario en su institución.	1		2		3		4		5		6	
72. La participación de los profesionales en actividades de educación continua.	1		2		3		4		5		6	
73. La aceptación del tratamiento por parte del paciente.	1		2		3		4		5		6	
74. El tiempo que se requiere para implementar la GPC.	1		2		3		4		5		6	
75. El conocimiento de los métodos de la medicina basada en la evidencia.	1		2		3		4		5		6	
76. La extensión del documento de la GPC.	1		2		3		4		5		6	

Por favor califique, según su experiencia o su percepción, en qué grado las siguientes condiciones **facilitan** la implementación de la GPC.

	Totalmente en desacuerdo		Moderadamente en desacuerdo		Un poco en desacuerdo		Un poco de acuerdo		Moderadamente de acuerdo		Totalmente de acuerdo	
77. La forma en la que se presenta la información de la GPC (Gráficas, tablas, etc).	1		2		3		4		5		6	
78. La terminología de la GPC (Calidad de la evidencia y fuerza de las recomendaciones).	1		2		3		4		5		6	
79. La multidisciplinariedad de los desarrolladores de la guía.	1		2		3		4		5		6	
80. El seguimiento institucional a la aplicación de la GPC.	1		2		3		4		5		6	
81. La difusión de la GPC.	1		2		3		4		5		6	
82. La inclusión de la GPC en la inducción institucional de los profesionales.	1		2		3		4		5		6	
83. Trabajar en un hospital universitario.	1		2		3		4		5		6	
84. La credibilidad de las GPC nacionales.	1		2		3		4		5		6	
85. La formación en GPC que reciben los profesionales por parte de EPS e IPS.	1		2		3		4		5		6	
86. La variabilidad en la atención de los pacientes.	1		2		3		4		5		6	
87. La fragmentación en la prestación de servicios en salud.	1		2		3		4		5		6	
88. El nivel de ingresos de los pacientes.	1		2		3		4		5		6	
89. La aceptación de las recomendaciones de la GPC por parte del personal de la salud.	1		2		3		4		5		6	
90. El reconocimiento de la utilidad de la GPC por parte del personal de la salud.	1		2		3		4		5		6	

¿Qué otros factores considera usted como barreras para implementar la guía?:

____

gruporehabilitacionsalud@udea.edu.co. Teléfono: 219 64 75. Dirección: Calle 62 52-59, Sede de Investigación Universitaria -SIU Torre 1, oficina 313. Medellín, Colombia.
