# Supplementary material for: Development and validation of a questionnaire to identify barriers to the implementation of the Clinical Practice Guidelines for lower limb amputees in a middle‐income country
Source: Clin Public Health Guidel. 2024 Mar 19;1(1):e12010. doi: 10.1002/gin2.12010 (PMC13563403; doi:10.1002/gin2.12010)
Supplement: Supplementary file 3 [file GIN2-1-e12010-s003.pdf]

Correlation matrix

		p1	p2	p3	p4	p5	p6	p7	p8	p9	p10	p11	p12	p13	p14	p15	p16	p17
Correlation	p1	1,000	,341	,259	,250	,257	,301	,195	,223	,307	,370	,428	,330	,329	,242	,337	,263	,282
	p2	,341	1,000	,447	,124	,175	,280	,306	,391	,166	,259	,307	,292	,178	,365	,234	,475	,397
	p3	,259	,447	1,000	,215	,142	,412	,283	,302	,129	,241	,291	,270	,149	,388	,213	,350	,483
	p4	,250	,124	,215	1,000	,371	,263	,335	,276	,452	,361	,249	,161	,227	,184	,219	,184	,205
	p5	,257	,175	,142	,371	1,000	,358	,270	,324	,410	,311	,252	,274	,355	,222	,533	,241	,252
	p6	,301	,280	,412	,263	,358	1,000	,355	,277	,257	,348	,358	,272	,258	,342	,332	,329	,397
	p7	,195	,306	,283	,335	,270	,355	1,000	,474	,358	,255	,148	,188	,243	,299	,215	,307	,237
	p8	,223	,391	,302	,276	,324	,277	,474	1,000	,253	,236	,235	,333	,273	,406	,320	,424	,230
	p9	,307	,166	,129	,452	,410	,257	,358	,253	1,000	,573	,368	,298	,351	,169	,290	,172	,222
	p10	,370	,259	,241	,361	,311	,348	,255	,236	,573	1,000	,601	,456	,409	,298	,322	,262	,417
	p11	,428	,307	,291	,249	,252	,358	,148	,235	,368	,601	1,000	,429	,339	,299	,309	,295	,420
	p12	,330	,292	,270	,161	,274	,272	,188	,333	,298	,456	,429	1,000	,466	,365	,377	,325	,416
	p13	,329	,178	,149	,227	,355	,258	,243	,273	,351	,409	,339	,466	1,000	,366	,438	,247	,307
	p14	,242	,365	,388	,184	,222	,342	,299	,406	,169	,298	,299	,365	,366	1,000	,386	,577	,499
	p15	,337	,234	,213	,219	,533	,332	,215	,320	,290	,322	,309	,377	,438	,386	1,000	,329	,336
	p16	,263	,475	,350	,184	,241	,329	,307	,424	,172	,262	,295	,325	,247	,577	,329	1,000	,521
	p17	,282	,397	,483	,205	,252	,397	,237	,230	,222	,417	,420	,416	,307	,499	,336	,521	1,000
	p18	,296	,351	,391	,141	,154	,312	,054	,125	,190	,439	,426	,450	,237	,369	,299	,426	,634
	p19	,278	,154	,193	,255	,287	,287	,244	,269	,370	,382	,333	,449	,431	,276	,242	,256	,353
	p20	,345	,110	,245	,229	,233	,293	,102	,127	,324	,382	,338	,307	,298	,196	,264	,224	,334
	p21	,330	,263	,317	,176	,119	,303	,065	,061	,222	,386	,443	,342	,197	,309	,216	,287	,491
	p22	,323	,361	,344	,144	,161	,304	,087	,108	,249	,441	,454	,420	,213	,359	,253	,309	,508
	p23	,272	,236	,249	,311	,208	,273	,189	,044	,355	,463	,418	,316	,328	,317	,236	,330	,469
	p24	,246	,183	,200	,266	,342	,162	,220	,163	,439	,383	,324	,286	,385	,264	,398	,264	,328
	p25	,129	,080	-,021	,121	,254	,099	,088	,058	,220	,211	,134	,146	,252	,053	,256	,061	,113
	p26	,272	,075	,037	,105	,201	,127	,095	,140	,117	,143	,127	,327	,261	,188	,195	,109	,131
	p27	,141	-,002	-,052	,098	,206	,086	,084	,097	,162	,126	,111	,032	,179	,047	,192	,067	,098
	p28	,205	,139	,137	,059	,053	,164	,055	,074	,000	,082	,116	,185	,108	,141	,130	,167	,165
	p29	,173	,168	,128	,028	,095	,102	,097	,200	,064	,139	,131	,189	,179	,156	,174	,204	,200
	p30	,184	,089	,017	,083	,260	,167	,109	,121	,177	,213	,186	,211	,233	,147	,286	,169	,168
	p31	,124	,079	,097	,170	,240	,139	,096	,162	,218	,179	,220	,257	,212	,147	,176	,175	,224
	p32	,130	,189	,158	,026	,119	,197	,126	,195	-,024	,061	,129	,162	,169	,167	,231	,264	,232
	p33	,143	,067	,036	,090	,317	,124	,048	,083	,183	,080	,116	,112	,157	,070	,264	,103	,108
	p34	,102	,174	,158	,030	,094	,127	,098	,154	,062	,100	,078	,197	,110	,166	,182	,180	,221
	p35	,120	,147	,163	,026	,028	,124	,059	,130	,036	,133	,122	,136	,164	,149	,185	,165	,151
	p36	,137	,088	,064	-,014	-,090	,073	,048	-,003	,022	,068	,111	,119	,022	,074	-,029	,151	,137
	p37	,240	,060	,070	,048	-,035	,149	-,004	-,007	,052	,137	,195	,186	,076	,089	,067	,129	,200
	p38	,083	,084	,037	,089	,251	,137	,104	,111	,108	,087	,075	,056	,149	,099	,285	,119	,118
	p39	,029	,073	,031	,058	,212	,114	,084	,029	,126	,038	,070	,046	,140	,009	,239	,051	,081
	p40	,113	,079	,064	,102	,120	,074	,081	,075	,186	,157	,196	,185	,216	,133	,149	,095	,149
	p41	,048	-,009	-,058	,054	,260	,126	,127	,135	,152	,050	-,013	,096	,225	,081	,224	,035	-,002
	p42	,097	,151	,102	,013	,141	,158	,157	,194	,058	,056	,040	,127	,185	,116	,213	,131	,083

	p43	,114	,019	,090	,119	,151	,128	,078	,119	,186	,168	,247	,241	,214	,133	,154	,135	,200
	p44	,153	,137	,131	,008	,106	,148	,082	,169	,140	,109	,230	,261	,238	,236	,158	,219	,219
	p45	,091	,037	,018	,111	,237	,154	,131	,120	,216	,140	,138	,138	,246	,096	,274	,070	,098
	p46	,244	,092	,088	,084	,062	,154	,078	,135	,101	,160	,144	,257	,207	,178	,152	,111	,157
	p47	,206	,118	,115	,114	,080	,166	,108	,126	,177	,183	,190	,204	,176	,229	,122	,215	,223
	p48	,162	,132	,048	,082	,078	,121	,184	,194	,143	,105	,169	,204	,220	,176	,147	,211	,131
	p49	-,042	,109	,104	-,108	-,077	,006	-,025	,019	-,152	-,083	-,042	,098	-,028	,113	,060	,073	,096
	p50	,017	,080	,036	-,099	-,066	-,060	,045	-,079	-,111	-,131	,031	,002	-,037	,053	-,011	,142	,069
	p51	,055	-,006	,026	,093	-,067	,019	-,082	,019	-,018	,010	,032	,072	-,044	,037	-,015	,021	,085
	p52	,012	,096	,175	-,041	-,072	,005	-,054	-,006	-,107	-,043	,034	,103	,010	,094	,047	,032	,133
	p53	-,034	,004	-,027	-,064	,002	-,037	-,011	-,076	-,064	-,059	,006	-,021	,059	-,008	-,004	-,024	,009
	p54	,125	,075	,104	,001	-,043	,069	-,013	-,081	-,012	,041	,074	,120	-,004	,095	,031	,044	,063
	p55	,124	-,024	-,010	,079	,012	,125	,010	-,081	,096	,048	,137	,081	,111	,006	,012	,001	,026
	p56	,118	-,010	-,041	,100	,041	,107	-,002	-,086	,037	,075	,066	,129	,100	,090	,039	-,003	,101
	p57	,044	,032	,033	,097	,004	,029	-,026	-,016	,009	-,002	,047	,099	-,021	,045	,039	,073	,062
	p58	,004	,050	,009	,089	,112	-,024	,077	,039	,120	-,039	-,045	-,087	,009	-,071	,122	-,005	-,034
	p59	,128	,042	,056	,184	,201	,121	,075	,044	,067	,040	,054	-,023	,020	-,065	,150	,023	,006
	p60	,174	,063	,109	,079	,033	,079	-,013	-,055	-,022	,091	,094	,156	,039	,079	,056	,016	,109
	p61	,187	,086	,114	,083	,001	,086	,038	-,026	-,017	,040	,103	,183	,063	,155	,073	,061	,161
	p62	,114	,110	,057	,068	,068	,137	,042	,097	,054	,085	,052	,118	,025	,053	,044	,064	,096
	p63	,085	,117	,097	,055	,075	,155	,098	,089	-,065	,010	-,010	,043	,026	,134	,060	,109	,107
	p64	,051	,082	,016	,041	-,002	-,001	,070	,053	,037	-,007	,002	,040	,107	,052	,013	,121	,085
	p65	,096	,099	,071	,031	-,019	,029	,012	,089	,053	,020	,018	,109	,054	,072	,063	,119	,117
	p66	-,091	-,005	,013	-,068	-,058	-,028	-,031	-,043	-,045	-,061	,013	-,085	-,044	,038	-,040	,004	-,031

Correlation matrix

		p18	p19	p20	p21	p22	p23	p24	p25	p26	p27	p28	p29	p30	p31	p32	p33	p34
Correlation	p1	,296	,278	,345	,330	,323	,272	,246	,129	,272	,141	,205	,173	,184	,124	,130	,143	,102
	p2	,351	,154	,110	,263	,361	,236	,183	,080	,075	-,002	,139	,168	,089	,079	,189	,067	,174
	p3	,391	,193	,245	,317	,344	,249	,200	-,021	,037	-,052	,137	,128	,017	,097	,158	,036	,158
	p4	,141	,255	,229	,176	,144	,311	,266	,121	,105	,098	,059	,028	,083	,170	,026	,090	,030
	p5	,154	,287	,233	,119	,161	,208	,342	,254	,201	,206	,053	,095	,260	,240	,119	,317	,094
	p6	,312	,287	,293	,303	,304	,273	,162	,099	,127	,086	,164	,102	,167	,139	,197	,124	,127
	p7	,054	,244	,102	,065	,087	,189	,220	,088	,095	,084	,055	,097	,109	,096	,126	,048	,098
	p8	,125	,269	,127	,061	,108	,044	,163	,058	,140	,097	,074	,200	,121	,162	,195	,083	,154
	p9	,190	,370	,324	,222	,249	,355	,439	,220	,117	,162	,000	,064	,177	,218	-,024	,183	,062
	p10	,439	,382	,382	,386	,441	,463	,383	,211	,143	,126	,082	,139	,213	,179	,061	,080	,100
	p11	,426	,333	,338	,443	,454	,418	,324	,134	,127	,111	,116	,131	,186	,220	,129	,116	,078
	p12	,450	,449	,307	,342	,420	,316	,286	,146	,327	,032	,185	,189	,211	,257	,162	,112	,197
	p13	,237	,431	,298	,197	,213	,328	,385	,252	,261	,179	,108	,179	,233	,212	,169	,157	,110
	p14	,369	,276	,196	,309	,359	,317	,264	,053	,188	,047	,141	,156	,147	,147	,167	,070	,166

p15	,299	,242	,264	,216	,253	,236	,398	,256	,195	,192	,130	,174	,286	,176	,231	,264	,182
p16	,426	,256	,224	,287	,309	,330	,264	,061	,109	,067	,167	,204	,169	,175	,264	,103	,180
p17	,634	,353	,334	,491	,508	,469	,328	,113	,131	,098	,165	,200	,168	,224	,232	,108	,221
p18	1,000	,336	,353	,577	,606	,493	,331	,127	,156	,049	,142	,231	,079	,109	,172	,044	,189
p19	,336	1,000	,449	,249	,243	,243	,260	,210	,262	,205	,167	,167	,266	,308	,168	,141	,095
p20	,353	,449	1,000	,502	,365	,311	,337	,175	,184	,144	,140	,116	,186	,265	,124	,109	,052
p21	,577	,249	,502	1,000	,723	,597	,375	,023	,150	,013	,138	,132	,092	,134	,139	,043	,144
p22	,606	,243	,365	,723	1,000	,614	,383	,060	,116	-,048	,117	,104	,062	,116	,140	,074	,143
p23	,493	,243	,311	,597	,614	1,000	,517	,119	,073	,012	,085	,070	,102	,120	,077	,048	,098
p24	,331	,260	,337	,375	,383	,517	1,000	,227	,059	,107	,036	,094	,150	,191	,080	,152	,134
p25	,127	,210	,175	,023	,060	,119	,227	1,000	,344	,429	,168	,231	,332	,243	,184	,322	,122
p26	,156	,262	,184	,150	,116	,073	,059	,344	1,000	,374	,489	,222	,352	,358	,238	,234	,118
p27	,049	,205	,144	,013	-,048	,012	,107	,429	,374	1,000	,329	,347	,466	,345	,339	,461	,120
p28	,142	,167	,140	,138	,117	,085	,036	,168	,489	,329	1,000	,257	,329	,324	,352	,213	,152
p29	,231	,167	,116	,132	,104	,070	,094	,231	,222	,347	,257	1,000	,397	,323	,367	,275	,465
p30	,079	,266	,186	,092	,062	,102	,150	,332	,352	,466	,329	,397	1,000	,496	,393	,403	,227
p31	,109	,308	,265	,134	,116	,120	,191	,243	,358	,345	,324	,323	,496	1,000	,300	,408	,251
p32	,172	,168	,124	,139	,140	,077	,080	,184	,238	,339	,352	,367	,393	,300	1,000	,327	,344
p33	,044	,141	,109	,043	,074	,048	,152	,322	,234	,461	,213	,275	,403	,408	,327	1,000	,276
p34	,189	,095	,052	,144	,143	,098	,134	,122	,118	,120	,152	,465	,227	,251	,344	,276	1,000
p35	,120	,076	,098	,012	,029	,012	,091	,111	,122	,138	,159	,311	,231	,272	,272	,222	,502
p36	,132	,028	,101	,191	,185	,173	,012	-,012	,126	,081	,197	,170	,143	,125	,291	,056	,165
p37	,207	,131	,194	,250	,192	,095	,048	,004	,221	,036	,247	,178	,174	,141	,139	,048	,176
p38	,061	,094	,082	,023	,044	,013	,122	,206	,123	,353	,208	,261	,327	,178	,408	,365	,313
p39	,026	,114	,135	,003	,026	,023	,139	,325	,229	,409	,233	,296	,427	,347	,271	,439	,228
p40	,072	,229	,172	,099	,071	,122	,085	,216	,321	,193	,262	,243	,408	,470	,182	,211	,150
p41	-,075	,146	,066	-,084	-,053	-,054	,090	,342	,268	,426	,231	,212	,509	,381	,332	,500	,194
p42	,027	,096	,104	,051	,053	-,004	,104	,148	,198	,279	,316	,361	,323	,257	,334	,297	,338
p43	,090	,323	,252	,087	,031	,070	,112	,216	,356	,218	,255	,212	,356	,540	,178	,212	,162
p44	,085	,244	,105	,044	,060	,088	,081	,163	,366	,189	,320	,175	,290	,382	,236	,180	,148
p45	,038	,179	,149	,005	,037	,019	,146	,344	,253	,375	,146	,284	,475	,377	,269	,434	,255
p46	,117	,186	,158	,086	,082	,051	,024	,159	,436	,195	,528	,177	,280	,256	,186	,174	,136
p47	,213	,189	,179	,190	,131	,203	,074	,194	,378	,232	,432	,249	,284	,311	,203	,139	,154
p48	,065	,242	,129	,064	,060	,036	,088	,222	,304	,271	,303	,270	,345	,364	,290	,228	,157
p49	,044	-,051	-,026	-,003	,045	-,023	-,023	-,064	,016	-,099	,003	,028	-,031	-,017	,065	,007	,035
p50	,041	-,005	-,002	,076	,026	,003	-,016	-,134	,010	-,041	,019	-,066	-,090	-,070	,027	-,033	-,099
p51	,099	,028	,066	,088	,093	,079	-,037	-,032	,103	-,006	,051	-,018	-,030	-,004	-,077	-,032	-,170
p52	,155	-,011	-,019	,075	,111	,043	-,001	-,115	,019	-,159	,053	,026	-,054	-,065	,042	-,104	,041
p53	-,046	-,015	-,106	-,112	-,102	-,017	-,038	,011	,096	,015	,054	-,084	,054	-,008	-,006	-,029	-,067
p54	,062	,006	,089	,123	,137	,053	,022	,010	,115	-,031	,140	-,024	,024	,001	,093	-,025	,043
p55	-,028	,104	,129	,113	,087	,150	,023	,012	,158	,064	,152	-,060	,030	,046	,003	-,002	-,015
p56	,063	,099	,044	,060	,048	,126	,015	,060	,351	,142	,214	-,063	,093	,099	,033	,097	-,087

	p57	,094	,097	,094	,029	,065	,045	,035	,020	,188	-,018	,148	-,033	,007	,076	-,014	-,050	-,092
	p58	-,078	,000	-,131	-,125	-,117	-,048	,058	,040	-,023	,116	,020	,045	,114	,063	,055	,204	,029
	p59	-,064	,041	,032	,023	,027	,028	,089	,114	,069	,177	,148	-,006	,131	,050	,192	,168	-,017
	p60	,092	,066	,140	,151	,138	,121	,007	-,036	,185	,000	,131	,011	,034	,058	,021	,007	,051
	p61	,092	,129	,123	,151	,141	,115	,025	,023	,222	-,002	,193	,029	,079	,075	,059	-,039	,074
	p62	,130	,077	,095	,111	,110	,080	,060	,039	,058	,124	,087	-,006	,139	,091	,168	,012	,055
	p63	,034	,090	,069	,102	,086	,071	-,024	-,113	,083	,050	,054	-,028	,009	,043	-,017	,006	,083
	p64	-,016	,086	-,018	-,022	-,058	-,006	,079	,046	,173	,119	,078	,011	,058	,110	,079	,090	,005
	p65	,086	,022	,034	,039	,002	,050	-,017	,031	,155	,036	,117	-,016	,057	,027	,047	,026	-,012
	p66	-,026	-,018	-,099	-,036	-,037	-,048	-,011	,036	-,047	-,012	-,021	-,004	,027	,007	-,050	,062	-,076

Correlation matrix

		p35	p36	p37	p38	p39	p40	p41	p42	p43	p44	p45	p46	p47	p48	p49	p50	p51
Correlation	p1	,120	,137	,240	,083	,029	,113	,048	,097	,114	,153	,091	,244	,206	,162	-,042	,017	,055
	p2	,147	,088	,060	,084	,073	,079	-,009	,151	,019	,137	,037	,092	,118	,132	,109	,080	-,006
	p3	,163	,064	,070	,037	,031	,064	-,058	,102	,090	,131	,018	,088	,115	,048	,104	,036	,026
	p4	,026	-,014	,048	,089	,058	,102	,054	,013	,119	,008	,111	,084	,114	,082	-,108	-,099	,093
	p5	,028	-,090	-,035	,251	,212	,120	,260	,141	,151	,106	,237	,062	,080	,078	-,077	-,066	-,067
	p6	,124	,073	,149	,137	,114	,074	,126	,158	,128	,148	,154	,154	,166	,121	,006	-,060	,019
	p7	,059	,048	-,004	,104	,084	,081	,127	,157	,078	,082	,131	,078	,108	,184	-,025	,045	-,082
	p8	,130	-,003	-,007	,111	,029	,075	,135	,194	,119	,169	,120	,135	,126	,194	,019	-,079	,019
	p9	,036	,022	,052	,108	,126	,186	,152	,058	,186	,140	,216	,101	,177	,143	-,152	-,111	-,018
	p10	,133	,068	,137	,087	,038	,157	,050	,056	,168	,109	,140	,160	,183	,105	-,083	-,131	,010
	p11	,122	,111	,195	,075	,070	,196	-,013	,040	,247	,230	,138	,144	,190	,169	-,042	,031	,032
	p12	,136	,119	,186	,056	,046	,185	,096	,127	,241	,261	,138	,257	,204	,204	,098	,002	,072
	p13	,164	,022	,076	,149	,140	,216	,225	,185	,214	,238	,246	,207	,176	,220	-,028	-,037	-,044
	p14	,149	,074	,089	,099	,009	,133	,081	,116	,133	,236	,096	,178	,229	,176	,113	,053	,037
	p15	,185	-,029	,067	,285	,239	,149	,224	,213	,154	,158	,274	,152	,122	,147	,060	-,011	-,015
	p16	,165	,151	,129	,119	,051	,095	,035	,131	,135	,219	,070	,111	,215	,211	,073	,142	,021
	p17	,151	,137	,200	,118	,081	,149	-,002	,083	,200	,219	,098	,157	,223	,131	,096	,069	,085
	p18	,120	,132	,207	,061	,026	,072	-,075	,027	,090	,085	,038	,117	,213	,065	,044	,041	,099
	p19	,076	,028	,131	,094	,114	,229	,146	,096	,323	,244	,179	,186	,189	,242	-,051	-,005	,028
	p20	,098	,101	,194	,082	,135	,172	,066	,104	,252	,105	,149	,158	,179	,129	-,026	-,002	,066
	p21	,012	,191	,250	,023	,003	,099	-,084	,051	,087	,044	,005	,086	,190	,064	-,003	,076	,088
	p22	,029	,185	,192	,044	,026	,071	-,053	,053	,031	,060	,037	,082	,131	,060	,045	,026	,093
	p23	,012	,173	,095	,013	,023	,122	-,054	-,004	,070	,088	,019	,051	,203	,036	-,023	,003	,079
	p24	,091	,012	,048	,122	,139	,085	,090	,104	,112	,081	,146	,024	,074	,088	-,023	-,016	-,037
	p25	,111	-,012	,004	,206	,325	,216	,342	,148	,216	,163	,344	,159	,194	,222	-,064	-,134	-,032
	p26	,122	,126	,221	,123	,229	,321	,268	,198	,356	,366	,253	,436	,378	,304	,016	,010	,103
	p27	,138	,081	,036	,353	,409	,193	,426	,279	,218	,189	,375	,195	,232	,271	-,099	-,041	-,006
	p28	,159	,197	,247	,208	,233	,262	,231	,316	,255	,320	,146	,528	,432	,303	,003	,019	,051
	p29	,311	,170	,178	,261	,296	,243	,212	,361	,212	,175	,284	,177	,249	,270	,028	-,066	-,018

	p30	,231	,143	,174	,327	,427	,408	,509	,323	,356	,290	,475	,280	,284	,345	-,031	-,090	-,030
	p31	,272	,125	,141	,178	,347	,470	,381	,257	,540	,382	,377	,256	,311	,364	-,017	-,070	-,004
	p32	,272	,291	,139	,408	,271	,182	,332	,334	,178	,236	,269	,186	,203	,290	,065	,027	-,077
	p33	,222	,056	,048	,365	,439	,211	,500	,297	,212	,180	,434	,174	,139	,228	,007	-,033	-,032
	p34	,502	,165	,176	,313	,228	,150	,194	,338	,162	,148	,255	,136	,154	,157	,035	-,099	-,170
	p35	1,000	,202	,213	,295	,206	,219	,194	,386	,239	,209	,244	,202	,162	,209	-,031	-,090	-,136
	p36	,202	1,000	,390	,078	,054	,228	,060	,079	,178	,146	,052	,159	,260	,163	-,035	-,015	,057
	p37	,213	,390	1,000	,183	,184	,269	,033	,179	,250	,261	,099	,315	,229	,251	,004	,019	,033
	p38	,295	,078	,183	1,000	,449	,165	,426	,455	,194	,201	,416	,255	,141	,247	-,061	-,091	-,141
	p39	,206	,054	,184	,449	1,000	,332	,531	,360	,273	,278	,451	,230	,178	,363	-,045	-,098	-,095
	p40	,219	,228	,269	,165	,332	1,000	,305	,229	,596	,438	,285	,373	,476	,403	-,023	-,122	-,022
	p41	,194	,060	,033	,426	,531	,305	1,000	,457	,265	,277	,516	,289	,174	,378	,023	-,119	-,109
	p42	,386	,079	,179	,455	,360	,229	,457	1,000	,299	,271	,369	,291	,203	,300	,022	-,019	-,134
	p43	,239	,178	,250	,194	,273	,596	,265	,299	1,000	,639	,358	,404	,485	,469	-,019	-,056	,022
	p44	,209	,146	,261	,201	,278	,438	,277	,271	,639	1,000	,364	,477	,503	,501	-,001	,005	-,006
	p45	,244	,052	,099	,416	,451	,285	,516	,369	,358	,364	1,000	,255	,163	,378	-,051	-,044	-,071
	p46	,202	,159	,315	,255	,230	,373	,289	,291	,404	,477	,255	1,000	,578	,398	-,011	-,064	,037
	p47	,162	,260	,229	,141	,178	,476	,174	,203	,485	,503	,163	,578	1,000	,448	-,042	-,074	,080
	p48	,209	,163	,251	,247	,363	,403	,378	,300	,469	,501	,378	,398	,448	1,000	-,022	-,026	-,009
	p49	-,031	-,035	,004	-,061	-,045	-,023	,023	,022	-,019	-,001	-,051	-,011	-,042	-,022	1,000	,279	,183
	p50	-,090	-,015	,019	-,091	-,098	-,122	-,119	-,019	-,056	,005	-,044	-,064	-,074	-,026	,279	1,000	,194
	p51	-,136	,057	,033	-,141	-,095	-,022	-,109	-,134	,022	-,006	-,071	,037	,080	-,009	,183	,194	1,000
	p52	,033	,053	,072	-,073	-,113	-,075	-,110	-,014	-,039	-,017	-,068	,131	,019	,032	,338	,299	,351
	p53	-,017	-,071	-,112	,007	-,065	-,010	,004	-,046	,050	,096	-,007	,067	-,021	,003	,139	,290	,100
	p54	,021	,162	,134	,001	-,016	,095	-,051	-,035	,115	,096	-,049	,131	,087	,082	,253	,214	,147
	p55	,004	,199	,204	,047	,037	,218	-,015	,026	,216	,154	-,016	,169	,158	,105	,109	,144	,067
	p56	,021	,079	,106	-,052	,038	,157	,036	,004	,231	,227	,032	,283	,262	,139	-,002	,059	,159
	p57	-,087	,052	,131	-,099	,060	,046	-,097	-,120	,087	,097	-,068	,073	,088	,089	,057	,067	,375
	p58	-,043	-,133	-,103	,180	,166	,057	,191	,104	,012	,057	,128	-,002	,017	,083	-,040	,026	-,039
	p59	-,040	,095	-,034	,194	,190	,072	,207	,053	,077	,040	,150	,090	,071	,151	-,042	-,015	,088
	p60	,077	,206	,172	-,044	-,044	,114	-,075	,017	,191	,130	-,021	,194	,128	,064	,196	,147	,168
	p61	,006	,154	,218	-,041	-,028	,196	-,054	,033	,241	,208	,011	,276	,227	,140	,141	,089	,202
	p62	,004	,041	,018	,060	-,022	,047	,072	,024	,019	,018	,004	,094	,112	,002	-,075	-,038	,005
	p63	,003	,092	,122	,032	,010	,046	-,018	,078	,139	,034	-,040	,072	,100	,093	,221	,124	,151
	p64	-,045	,018	-,033	-,018	,054	,155	,038	,008	,174	,108	,056	,052	,157	,170	,068	,198	,100
	p65	,028	-,026	,053	,016	-,008	,101	,011	-,023	,092	,095	,051	,184	,120	,137	,153	,104	,185
	p66	-,062	-,082	-,015	-,114	-,029	-,024	,027	-,074	-,088	-,032	-,007	-,057	-,116	-,012	,034	,050	,068

Correlation matrix

		p52	p53	p54	p55	p56	p57	p58	p59	p60	p61	p62	p63	p64	p65	p66
Correlation	p1	,012	-,034	,125	,124	,118	,044	,004	,128	,174	,187	,114	,085	,051	,096	-,091
	p2	,096	,004	,075	-,024	-,010	,032	,050	,042	,063	,086	,110	,117	,082	,099	-,005

p3	,175	-,027	,104	-,010	-,041	,033	,009	,056	,109	,114	,057	,097	,016	,071	,013
p4	-,041	-,064	,001	,079	,100	,097	,089	,184	,079	,083	,068	,055	,041	,031	-,068
p5	-,072	,002	-,043	,012	,041	,004	,112	,201	,033	,001	,068	,075	-,002	-,019	-,058
p6	,005	-,037	,069	,125	,107	,029	-,024	,121	,079	,086	,137	,155	-,001	,029	-,028
p7	-,054	-,011	-,013	,010	-,002	-,026	,077	,075	-,013	,038	,042	,098	,070	,012	-,031
p8	-,006	-,076	-,081	-,081	-,086	-,016	,039	,044	-,055	-,026	,097	,089	,053	,089	-,043
p9	-,107	-,064	-,012	,096	,037	,009	,120	,067	-,022	-,017	,054	-,065	,037	,053	-,045
p10	-,043	-,059	,041	,048	,075	-,002	-,039	,040	,091	,040	,085	,010	-,007	,020	-,061
p11	,034	,006	,074	,137	,066	,047	-,045	,054	,094	,103	,052	-,010	,002	,018	,013
p12	,103	-,021	,120	,081	,129	,099	-,087	-,023	,156	,183	,118	,043	,040	,109	-,085
p13	,010	,059	-,004	,111	,100	-,021	,009	,020	,039	,063	,025	,026	,107	,054	-,044
p14	,094	-,008	,095	,006	,090	,045	-,071	-,065	,079	,155	,053	,134	,052	,072	,038
p15	,047	-,004	,031	,012	,039	,039	,122	,150	,056	,073	,044	,060	,013	,063	-,040
p16	,032	-,024	,044	,001	-,003	,073	-,005	,023	,016	,061	,064	,109	,121	,119	,004
p17	,133	,009	,063	,026	,101	,062	-,034	,006	,109	,161	,096	,107	,085	,117	-,031
p18	,155	-,046	,062	-,028	,063	,094	-,078	-,064	,092	,092	,130	,034	-,016	,086	-,026
p19	-,011	-,015	,006	,104	,099	,097	,000	,041	,066	,129	,077	,090	,086	,022	-,018
p20	-,019	-,106	,089	,129	,044	,094	-,131	,032	,140	,123	,095	,069	-,018	,034	-,099
p21	,075	-,112	,123	,113	,060	,029	-,125	,023	,151	,151	,111	,102	-,022	,039	-,036
p22	,111	-,102	,137	,087	,048	,065	-,117	,027	,138	,141	,110	,086	-,058	,002	-,037
p23	,043	-,017	,053	,150	,126	,045	-,048	,028	,121	,115	,080	,071	-,006	,050	-,048
p24	-,001	-,038	,022	,023	,015	,035	,058	,089	,007	,025	,060	-,024	,079	-,017	-,011
p25	-,115	,011	,010	,012	,060	,020	,040	,114	-,036	,023	,039	-,113	,046	,031	,036
p26	,019	,096	,115	,158	,351	,188	-,023	,069	,185	,222	,058	,083	,173	,155	-,047
p27	-,159	,015	-,031	,064	,142	-,018	,116	,177	,000	-,002	,124	,050	,119	,036	-,012
p28	,053	,054	,140	,152	,214	,148	,020	,148	,131	,193	,087	,054	,078	,117	-,021
p29	,026	-,084	-,024	-,060	-,063	-,033	,045	-,006	,011	,029	-,006	-,028	,011	-,016	-,004
p30	-,054	,054	,024	,030	,093	,007	,114	,131	,034	,079	,139	,009	,058	,057	,027
p31	-,065	-,008	,001	,046	,099	,076	,063	,050	,058	,075	,091	,043	,110	,027	,007
p32	,042	-,006	,093	,003	,033	-,014	,055	,192	,021	,059	,168	-,017	,079	,047	-,050
p33	-,104	-,029	-,025	-,002	,097	-,050	,204	,168	,007	-,039	,012	,006	,090	,026	,062
p34	,041	-,067	,043	-,015	-,087	-,092	,029	-,017	,051	,074	,055	,083	,005	-,012	-,076
p35	,033	-,017	,021	,004	,021	-,087	-,043	-,040	,077	,006	,004	,003	-,045	,028	-,062
p36	,053	-,071	,162	,199	,079	,052	-,133	,095	,206	,154	,041	,092	,018	-,026	-,082
p37	,072	-,112	,134	,204	,106	,131	-,103	-,034	,172	,218	,018	,122	-,033	,053	-,015
p38	-,073	,007	,001	,047	-,052	-,099	,180	,194	-,044	-,041	,060	,032	-,018	,016	-,114
p39	-,113	-,065	-,016	,037	,038	,060	,166	,190	-,044	-,028	-,022	,010	,054	-,008	-,029
p40	-,075	-,010	,095	,218	,157	,046	,057	,072	,114	,196	,047	,046	,155	,101	-,024
p41	-,110	,004	-,051	-,015	,036	-,097	,191	,207	-,075	-,054	,072	-,018	,038	,011	,027
p42	-,014	-,046	-,035	,026	,004	-,120	,104	,053	,017	,033	,024	,078	,008	-,023	-,074
p43	-,039	,050	,115	,216	,231	,087	,012	,077	,191	,241	,019	,139	,174	,092	-,088
p44	-,017	,096	,096	,154	,227	,097	,057	,040	,130	,208	,018	,034	,108	,095	-,032

p45	-,068	-,007	-,049	-,016	,032	-,068	,128	,150	-,021	,011	,004	-,040	,056	,051	-,007
p46	,131	,067	,131	,169	,283	,073	-,002	,090	,194	,276	,094	,072	,052	,184	-,057
p47	,019	-,021	,087	,158	,262	,088	,017	,071	,128	,227	,112	,100	,157	,120	-,116
p48	,032	,003	,082	,105	,139	,089	,083	,151	,064	,140	,002	,093	,170	,137	-,012
p49	,338	,139	,253	,109	-,002	,057	-,040	-,042	,196	,141	-,075	,221	,068	,153	,034
p50	,299	,290	,214	,144	,059	,067	,026	-,015	,147	,089	-,038	,124	,198	,104	,050
p51	,351	,100	,147	,067	,159	,375	-,039	,088	,168	,202	,005	,151	,100	,185	,068
p52	1,000	,221	,277	,110	,102	,228	-,070	,027	,283	,278	-,039	,212	,104	,169	,013
p53	,221	1,000	,238	,263	,265	,098	,190	,079	,151	,157	-,072	,150	,328	,217	,066
p54	,277	,238	1,000	,524	,282	,160	-,021	,239	,448	,408	-,004	,285	,187	,164	-,039
p55	,110	,263	,524	1,000	,388	,154	-,011	,233	,407	,354	-,026	,370	,218	,214	-,108
p56	,102	,265	,282	,388	1,000	,319	,081	,139	,373	,502	-,005	,269	,271	,135	-,057
p57	,228	,098	,160	,154	,319	1,000	,049	,196	,222	,260	-,078	,239	,133	,145	,009
p58	-,070	,190	-,021	-,011	,081	,049	1,000	,343	-,026	-,020	-,104	,084	,289	,114	,107
p59	,027	,079	,239	,233	,139	,196	,343	1,000	,281	,203	,033	,204	,244	,131	-,052
p60	,283	,151	,448	,407	,373	,222	-,026	,281	1,000	,544	-,058	,383	,198	,173	-,131
p61	,278	,157	,408	,354	,502	,260	-,020	,203	,544	1,000	-,007	,331	,310	,169	-,041
p62	-,039	-,072	-,004	-,026	-,005	-,078	-,104	,033	-,058	-,007	1,000	-,061	-,016	-,049	-,247
p63	,212	,150	,285	,370	,269	,239	,084	,204	,383	,331	-,061	1,000	,270	,099	-,067
p64	,104	,328	,187	,218	,271	,133	,289	,244	,198	,310	-,016	,270	1,000	,255	,048
p65	,169	,217	,164	,214	,135	,145	,114	,131	,173	,169	-,049	,099	,255	1,000	,043
p66	,013	,066	-,039	-,108	-,057	,009	,107	-,052	-,131	-,041	-,247	-,067	,048	,043	1,000

Communalities

	Initial	Extraction
p1	1,000	,536
p2	1,000	,535
p3	1,000	,619
p4	1,000	,528
p5	1,000	,597
p6	1,000	,568
p7	1,000	,611
p8	1,000	,675
p9	1,000	,678
p10	1,000	,648
p11	1,000	,597
p12	1,000	,565
p13	1,000	,602
p14	1,000	,632
p15	1,000	,592

p16	1,000	,644
p17	1,000	,657
p18	1,000	,703
p19	1,000	,505
p20	1,000	,518
p21	1,000	,720
p22	1,000	,718
p23	1,000	,701
p24	1,000	,603
p25	1,000	,451
p26	1,000	,644
p27	1,000	,644
p28	1,000	,600
p29	1,000	,600
p30	1,000	,567
p31	1,000	,659
p32	1,000	,573
p33	1,000	,546
p34	1,000	,688
p35	1,000	,594
p36	1,000	,597
p37	1,000	,575
p38	1,000	,616
p39	1,000	,594
p40	1,000	,603
p41	1,000	,647
p42	1,000	,529
p43	1,000	,761
p44	1,000	,675
p45	1,000	,547
p46	1,000	,716
p47	1,000	,646
p48	1,000	,558
p49	1,000	,523
p50	1,000	,603
p51	1,000	,642
p52	1,000	,622
p53	1,000	,556
p54	1,000	,564
p55	1,000	,656
p56	1,000	,644
p57	1,000	,589

p58	1,000	,603
p59	1,000	,623
p60	1,000	,618
p61	1,000	,588
p62	1,000	,664
p63	1,000	,560
p64	1,000	,609
p65	1,000	,423
p66	1,000	,653

Extraction method: principal component analysis.

Matriz de componente[a]

	Componente														
	1	2	3	4	5	6	7	8	9	10	11	12	13	14	15
p17	,601	,412													
p12	,589														
p10	,559														
p11	,555														
p15	,553														
p31	,551														
p30	,551	-,424													
p13	,551														
p19	,547														
p43	,539														
p47	,523														
p14	,518														
p44	,517														
p16	,516														
p18	,514	,483													
p1	,509														

p26	,507														
p48	,506														
p6	,499														
p20	,499														
p46	,494														
p40	,492														
p9	,471														
p5	,467			,450											
p32	,460														
p24	,459														
p28	,458														
p29	,437														
p33	,423	-,419													
p2	,422														
p42	,417	-,405													
p3	,403														
p25															
p7															
p35															
p41	,406	-,595													
p22	,488	,530													
p39	,413	-,507													
p21	,479	,502													
p23	,473	,484													
p45	,463	-,466													
p27	,415	-,457													
p38		-,410													
p61			,639												
p60			,617												
p54			,587												

p55			,573												
p56			,566												
p63			,474												
p52			,439												
p64			,409												
p53			,409												
p59				,412											
p37															
p36															
p4															
p49					,415										
p50															
p8	,410					-,467									
p66							,427								
p62								,414							
p58				,439					,459						
p57			,428							,446					
p51										,401					
p34															
p65															

Extraction method: principal component analysis.

Varianza total explicada

Componente	Autovalores iniciales			Sumas de extracción de cargas al cuadrado		
	Total	% de varianza	% acumulado	Total	% de varianza	% acumulado
1	11,221	17,002	17,002	11,221	17,002	17,002
2	5,366	8,130	25,132	5,366	8,130	25,132
3	4,468	6,770	31,902	4,468	6,770	31,902
4	2,737	4,146	36,048	2,737	4,146	36,048
5	2,510	3,802	39,850	2,510	3,802	39,850
6	2,018	3,058	42,908	2,018	3,058	42,908
7	1,793	2,717	45,625	1,793	2,717	45,625
8	1,534	2,324	47,950	1,534	2,324	47,950
9	1,453	2,202	50,152	1,453	2,202	50,152
10	1,336	2,025	52,176	1,336	2,025	52,176
11	1,213	1,838	54,015	1,213	1,838	54,015
12	1,194	1,810	55,824	1,194	1,810	55,824
13	1,136	1,721	57,545	1,136	1,721	57,545
14	1,120	1,697	59,242	1,120	1,697	59,242
15	1,022	1,549	60,791	1,022	1,549	60,791
16	,995	1,507	62,298			
17	,976	1,479	63,777			
18	,940	1,425	65,201			
19	,907	1,374	66,575			
20	,862	1,305	67,880			
21	,857	1,298	69,178			
22	,838	1,270	70,449			
23	,797	1,208	71,657			
24	,785	1,190	72,847			
25	,755	1,145	73,991			
26	,725	1,098	75,090			
27	,698	1,057	76,147			
28	,672	1,018	77,165			
29	,651	,986	78,151			

30	,626	,948	79,100			
31	,617	,935	80,035			
32	,604	,915	80,950			
33	,595	,901	81,851			
34	,570	,864	82,715			
35	,564	,854	83,569			
36	,558	,845	84,414			
37	,548	,831	85,245			
38	,515	,780	86,025			
39	,496	,752	86,776			
40	,474	,718	87,494			
41	,458	,693	88,187			
42	,444	,673	88,861			
43	,431	,653	89,513			
44	,414	,627	90,140			
45	,412	,625	90,765			
46	,401	,607	91,372			
47	,390	,591	91,963			
48	,385	,583	92,546			
49	,367	,557	93,103			
50	,356	,539	93,642			
51	,334	,506	94,148			
52	,319	,483	94,631			
53	,314	,476	95,107			
54	,311	,471	95,578			
55	,306	,463	96,041			
56	,295	,447	96,488			
57	,277	,420	96,908			
58	,263	,399	97,307			
59	,260	,394	97,700			

60	,248	,376	98,076			
61	,244	,370	98,446			
62	,223	,338	98,784			
63	,216	,328	99,112			
64	,206	,312	99,424			
65	,194	,294	99,718			
66	,186	,282	100,000			

Extraction method: principal component analysis.
